# Bayes factors for superiority, non-inferiority, and equivalence designs

**DOI:** 10.1186/s12874-019-0699-7

**Published:** 2019-03-29

**Authors:** Don van Ravenzwaaij, Rei Monden, Jorge N. Tendeiro, John P. A. Ioannidis

**Affiliations:** 1 University of Groningen, Department of Psychology, Grote Kruisstraat 2/1, Heymans Building, Groningen, 9712 TS The Netherlands; 2 University Medical Center Groningen, Groningen, The Netherlands; 3 Departments of Medicine, of Health Research and Policy, of Biomedical Data Science, and of Statistics, and Meta-Research Innovation Center, Stanford, USA

**Keywords:** Bayes factors, Clinical trials, Statistical inference, Non-inferiority designs

## Abstract

**Background:**

In clinical trials, study designs may focus on assessment of superiority, equivalence, or non-inferiority, of a new medicine or treatment as compared to a control. Typically, evidence in each of these paradigms is quantified with a variant of the null hypothesis significance test. A null hypothesis is assumed (null effect, inferior by a specific amount, inferior by a specific amount *and* superior by a specific amount, for superiority, non-inferiority, and equivalence respectively), after which the probabilities of obtaining data more extreme than those observed under these null hypotheses are quantified by *p*-values. Although ubiquitous in clinical testing, the null hypothesis significance test can lead to a number of difficulties in interpretation of the results of the statistical evidence.

**Methods:**

We advocate quantifying evidence instead by means of Bayes factors and highlight how these can be calculated for different types of research design.

**Results:**

We illustrate Bayes factors in practice with reanalyses of data from existing published studies.

**Conclusions:**

Bayes factors for superiority, non-inferiority, and equivalence designs allow for explicit quantification of evidence in favor of the null hypothesis. They also allow for interim testing without the need to employ explicit corrections for multiple testing.

## Background

**Fig. 1:**
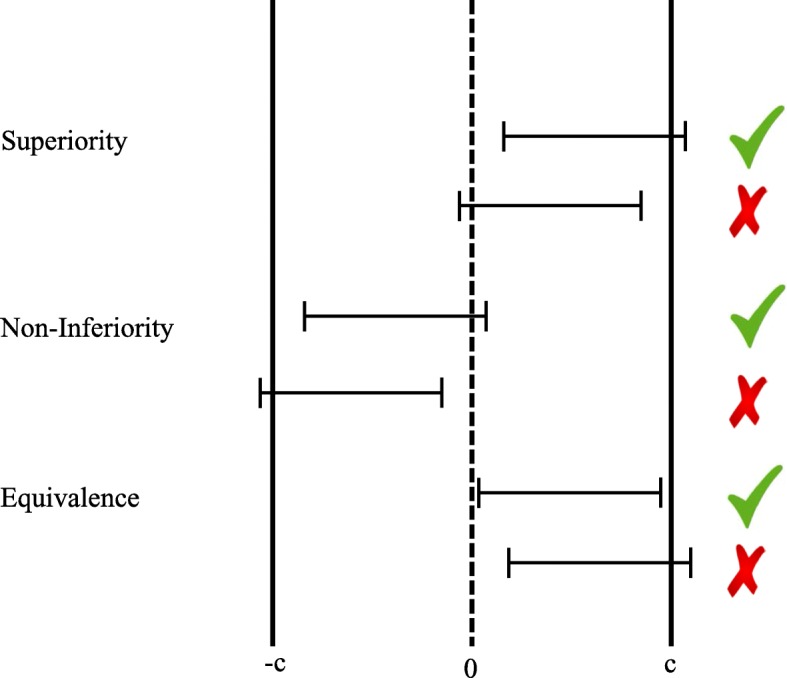
Superiority, non-inferiority, and equivalence designs. Each design type has an example for which the prospective null-hypothesis was rejected (the ones highlighted with check marks) and one for which the null-hypothesis was not rejected (the ones highlighted with fail marks). Error bars indicate 95% confidence intervals. Value −*c* is the null hypothesis value for non-inferiority testing, values −*c* and *c* are the two null hypotheses values for equivalence testing

In clinical trials, study designs may focus on assessment of superiority, equivalence, or non-inferiority of a new medicine or other intervention as compared to some control intervention [1, 2]. Typically, evidence in each of these paradigms is quantified with a variant of the null hypothesis significance test (NHST). A null hypothesis is assumed, after which the probability of obtaining data more extreme than those observed under the null hypothesis is quantified by a *p*-value. The specific null hypothesis that forms the basis of these tests differs depending on the design. A graphical display of each of the three designs is provided in Fig. 1.

The first, and by far most common, type of design is the superiority design (see top two rows). In the superiority design, the null hypothesis is that the true population effect size is exactly zero. The test can typically be conceived as being one-tailed, even though in practice superiority analyses often employ a two-tailed test. In other words, the null hypothesis states that a new medicine or other intervention being tested does not work better than an existing placebo or active control. The first row in Fig. 1 provides an example of a superiority design in which the null hypothesis was rejected and the second row in Fig. 1 provides an example of a superiority design in which the null hypothesis was not rejected.

The second type of design is the non-inferiority design (see middle two rows). In the non-inferiority design, the null hypothesis is that the true population effect size is lower than −*c*. This amounts to a one-tailed test in which a point-null hypothesis of effect size =−*c* is compared to an alternative hypothesis of effect size >−*c*. In other words, the relevant test is that a new medicine or other intervention being tested works better than an existing placebo or medication minus an apriori determined amount *c*. The third row in Fig. 1 provides an example of a non-inferiority design in which the null hypothesis was rejected and the fourth row in Fig. 1 provides an example of a non-inferiority design in which the null hypothesis was not rejected. Note that it is possible for an intervention to be deemed non-inferior, but simultaneously lower than zero (in this case, the constructed confidence interval would fall between −*c* and zero in its entirety).

The third type of design is the equivalence design (see bottom two rows). In the equivalence design, one essentially carries out two NHSTs. In this design, the null hypotheses are that the true population effect size is lower than −*cand* higher than *c*. This amounts to a one-tailed test in which a point-null hypothesis of effect size =−*c* is compared to an alternative hypothesis of effect size >−*cand* a one-tailed test in which a point-null hypothesis of effect size =*c* is compared to an alternative hypothesis of effect size <*c*. If both of these null hypotheses are rejected, equivalence is established. Graphically speaking, equivalence is established if the confidence interval falls in its entirety between the borders of −*c* and *c*. The fifth row in Fig. 1 provides an example of an equivalence design in which both null hypotheses were rejected and the sixth row in Fig. 1 provides an example of an equivalence design in which at least one of the two null hypotheses was not rejected. Analogous to non-inferiority designs, it is possible for an intervention to be deemed equivalent, but simultaneously different from zero (in this case, the constructed confidence interval would either fall between −*c* and zero in its entirety or between zero and *c* in its entirety). Study results can be interpreted very differently depending on whether the original design was a superiority or an equivalence design [3].

Each of these designs seeks to answer important questions. Unfortunately, the NHSTs employed to carry out statistical inference do not allow researchers to quantify evidence in favor of the null hypothesis. The desire to quantify evidence in favor of the null hypothesis is perhaps most relevant in equivalence designs. We quote Greene, Concato, and Feinstein [4], who say: “…Methodological flaws in a systematic review of 88 studies claiming equivalence, published from 1992 to 1996. Equivalence was inappropriately claimed in 67% of them, on the basis of nonsignificant tests for superiority. Fifty-one percent stated equivalence as an aim, but only 23% were designed with a preset margin of equivalence. Only 22% adopted appropriate practice: a predefined aim of equivalence, a preset *Δ*, consequent sample size determination, and actually testing equivalence.” A non-significant *p*-value (any *p*>.05) can result from (1) the null hypothesis being true or (2) the null hypothesis being false combined with an underpowered trial (that is, if we would have collected more data, the results of our inference would have been statistically significant see [5]. In medical research, it is important to distinguish between these two scenarios. Quantifying evidence in favor of the null hypothesis potentially leads to a reduction in the waste of scarce research resources, as research into ineffectual interventions can be discontinued [6].

Another problem with NHST emerges when there is multiple testing in interim analyses. In biomedicine, a range of methods exists that are employed to account for sequential testing and interim analyses, and they all basically change the level of statistical significance by asking for more stringent statistical thresholds to reject the null hypotheses when multiple analyses due to sequential testing or interim re-assessments are performed. However, these correction methods are not always applied. Furthermore, the number of participants tested in clinical trials often changes relative to the number decided upon a-priori based on interim analysis results [7]. Both of these practices lead to an overestimation of the evidence in favor of an effect.

Bayesian methods are an alternative to NHST that allow quantification of evidence in favor of the null hypothesis, sequential testing, and comparison of strength of evidence across different studies [8, 9]. Bayesian methods are increasingly considered for more widespread use in clinical trials (see e.g., [10]; for an overview of different fields, see [11]) and their advantages have been argued many times (e.g., [12–17], but see [18]). Several approaches to carrying out Bayesian inference exist, but for the remainder of this manuscript we will focus on the *Bayes factor* [19, 20]. The Bayes factor allows for explicit quantification of evidence in favor of the null hypothesis, which means that the interpretational pitfalls associated with non-inferiority and equivalence designs naturally disappear.

In the case of equivalance designs, traditional methods require specification of a potentially arbitrary band around zero, even if clear theoretical grounds for the width of this band are lacking. Bayes factors can quantify evidence in favor of a point null hypothesis or in favor of an interval null hypothesis, depending on which one is theoretically appropriate.

Bayes factors also allow for sequential testing without having to correct for multiple testing (see e.g. the simulation results reported in [21]). It is “…entirely appropriate to collect data until a point has been proven or disproven, or until the data collector runs out of time, money, or patience” [22], but see [23]. To put this quote into perspective, NHST has essentially one decision criterion (i.e., *p*<*α*). As such, if one employs sequential testing, every additional test increases the chance that this criterion is reached, even if the null hypothesis is true (see Table 1 in [24]). Bayesian testing does not require a fixed *n* in the sampling plan because the decision criterion is symmetrical. If one were to decide, for instance, to test until the relative evidence for one hypothesis over the other is at least ten, one would stop when the evidence provided by the data is ten over one in favor of the alternative hypothesis *or* ten over one in favor of the null hypothesis, and one would be wrong once for every ten times one were correct. The Bayes factor will provide progressively stronger relative support for the hypothesis that is true when data continues to be collected.

In what follows, we will describe how to implement Bayes factors for the three types of study design mentioned above.

## Methods

### The Bayes factor

Bayesian statisticians use probability distributions to quantify uncertainty or degree of belief about statistical propositions [25, 26]. For a given statistical model, say *M*, the prior distribution or prior *p*(*θ*|*M*) for a parameter *θ* is updated after encountering data *y* to yield a posterior distribution or posterior *p*(*θ*|*y*,*M*). Bayesian statistics can be viewed as a method for the rational updating of beliefs about statistical propositions. Specifically, Bayes’ rule combines the prior, what we believe to be true before having seen the data, with the likelihood, what the data tell us we should believe about the data, to obtain the posterior, what we believe to be true after having seen the data:

1 $$ p(\theta|y,M)= \frac{p(\theta|M)p(y|\theta,M)}{p(y|M)}= \frac{prior \times likelihood}{marginal~likelihood}   $$

In this equation, *p*(*y*|*M*) is the marginal likelihood of the data, a constant that does not involve *θ*. The posterior *p*(*θ*|*y*,*M*) is a mathematical product of prior knowledge *p*(*θ*|*M*) and the information coming from the data *p*(*y*|*θ*,*M*); hence, the posterior contains all that we know about *θ* (under model *M*) after observing the data *y*.

A similar Bayesian procedure can be used for hypothesis testing. Consider for example the choice between hypotheses *H*_0_ (the null hypothesis) and *H*_1_ (the alternative hypothesis). Bayes’ theorem dictates how the prior probability of *H*_0_, *p*(*H*_0_), is updated through the data to give the posterior probability of *H*_0_:

2 $$ p(H_{0}|y)= \frac{p(H_{0})p(y|H_{0})}{p(H_{0})p(y|H_{0})+p(H_{1})p(y|H_{1})}   $$

In the same way, one can calculate the posterior probability of *H*_1_, *p*(*H*_1_|*y*). These quantities require specification of the null hypothesis *H*_0_ and the alternative hypothesis *H*_1_. A common choice is to specify the hypotheses in terms of effect size [27]. The null hypothesis then becomes *H*_0_:*δ*=0 and the alternative hypothesis becomes *H*_1_:*δ*≠0 (or, alternatively, *H*_1_:*δ*<0 or *H*_1_:*δ*>0).

The ratio of the posterior probabilities is given by

3 $$ \frac{p(H_{1}|y)}{p(H_{0}|y)} = \frac{p(H_{1})}{p(H_{0})} \times \frac{p(y|H_{1})}{p(y|H_{0})}   $$

which shows that the change from prior odds of the hypotheses *p*(*H*_1_)/*p*(*H*_0_) to posterior odds of the hypotheses *p*(*H*_1_|*y*)/*p*(*H*_0_|*y*) is given by the ratio of marginal likelihoods *p*(*y*|*H*_1_)/*p*(*y*|*H*_0_), a quantity known as the Bayes factor, or BF [19, 20].

To see how Bayes factors may be obtained for point null hypotheses, it is illustrative to first consider the calculation of a Bayes factor for interval hypotheses. Let *H*_0_ be that the population effect size falls in an interval around zero: −*c*<*δ*<*c* and let *H*_1_ be that the population effect size does not fall in that interval: *δ*<−*c* or *δ*>*c*. We obtain the Bayes factor by calculating (*p*(*H*1|*data*)/*p*(*H*1))/(*p*(*H*0)/*p*(*H*0|*data*)). The smaller one chooses *c* (and therefore the interval around zero), the more *p*(*H*0|*data*)/*p*(*H*0) will dominate in the calculation of the Bayes factor, as *p*(*H*1|*data*)/*p*(*H*1) will tend to 1. In the limit of a point null hypothesis, one can get the Bayes factor by calculating *p*(*H*0)/*p*(*H*0|*data*), or by evaluating the ratio of the density of the prior and the posterior, evaluated at *δ*=0. This way of calculating the Bayes factor for point null hypotheses is known as the Savage-Dickey procedure, see [28] for a mathematical proof. Alternatively, one could calculate Bayes factors for a point null hypothesis over a point alternative hypothesis (say *δ*=0.25), based on prior study results or theoretical grounds [23].

Bayes factors represent “the primary tool used in Bayesian inference for hypothesis testing and model selection” [29, p. 378]; Bayes factors allow researchers to quantify evidence in favor of the null hypothesis vis à vis the alternative hypothesis. For instance, when a Bayes factor *BF*_10_=10, with the subscript meaning the alternative hypothesis over the null hypothesis, the observed data are 10 times more likely to have occurred under the alternative hypothesis than under the null hypothesis. When *BF*_10_=1/10=0.1, the observed data are ten times more likely to have occurred under the null hypothesis than under the alternative hypothesis. As for interpreting the strength of evidence as quantified by a Bayes factor, an often-used standard is described in [30]. The authors classify a Bayes factor between 1 and 3 (or, conversely, between 1/3 and 1) as ‘not worth more than a bare mention’, a Bayes factor between 3 and 20 (or, conversely, between 1/20 and 1/3) as ‘positive’, and a Bayes factor between 20 and 150 (or, conversely, between 1/150 and 1/20) as ‘strong’.

Foundational work on choosing appropriate priors for calculating Bayes factors has been done by Jeffreys [19] and the resulting ‘default’ Bayes factor remains to this day one of the most popular approaches to obtaining Bayes factors. We will briefly describe the default Bayes factor, then discuss more recent extensions to this work [27, 31].

### The default Bayes factor and implementations

Jeffreys’ [19] work applies to situations where the two hypotheses to be compared break down into a hypothesis that assigns a single value to the parameter of interest and a hypothesis that specifies a range of values to the parameter of interest. In biomedicine, the practical analogue of this is a point null hypothesis that specifies *δ*=0, where *δ* is an effect size parameter, and an alternative hypothesis that may specify *δ*<0,*δ*>0, or *δ*≠0.

Jeffreys [19] chose a Cauchy prior distribution with location parameter 0 and scale parameter 1 for the effect size *δ* parameter. This choice was motivated by the fact that it led to a Bayes factor of exactly 1 in case of completely uninformative data, and on the fact that the Bayes factor would tend to infinity or 1/infinity when the data are overwhelmingly informative. Mathematically, this Cauchy prior corresponds to a normal prior with a mean *μ*_*δ*_ of zero and a variance *g* that itself follows a scaled inverse chi-square distribution with one degree of freedom, in which the variance is integrated out [32, 33]. It is important to note that Jeffreys’ choice of prior was largely motivated by practical reasons, he had no philosophical objections to more informed priors. An extensive discussion of desiderata related to the choice of objective prior distributions may be found in [34].

The impact of Jeffreys’ default Bayes factor had been mostly theoretical until quite recently. An online tool was developed to calculate default Bayes factors for diverse *t*-test designs ([27], available at http://pcl.missouri.edu/bayesfactor). This same group also created the BayesFactor package for the statistical freeware program R [35]. An alternative group, focusing more on informative hypothesis testing, developed the Bain package for the statistical freeware program R [36]. Specialized point–and–click computer software was created for the explicit purpose of doing Bayesian analyses [37] which incorporates many features from the BayesFactor and Bain packages.

In recent work, derivations and R code are provided for (among other things) shifting the center of the Cauchy distribution away from zero [31, code may be found at https://osf.io/bsp6z/]. The full equation for obtaining the Bayes factor of the alternative hypothesis *δ*≠0 relative to the null hypothesis *δ*=0,*BF*_10_, modified from Equation 13 by Gronau et al. [31], is given by

4 $$ {{BF}_{10} = \frac{\begin{aligned} \int_{0}^{\infty}(1+ng)^{-\frac{1}{2}} exp\left(-\frac{\mu_{\delta}^{2}}{2\left(\frac{1}{n}+g\right)}\right)\left(1+\frac{t^{2}}{(1+ng)(n-1)}\right)^{-\frac{n}{2}} \times \\ \Bigg[\Gamma\left(\frac{n}{2}\right) {}_{1}F_{1}\left(\frac{n}{2};\frac{1}{2};\frac{\mu_{\delta}^{2} t^{2}}{2\left(\frac{1}{n}+g\right)\left[\left(1+ng\right)\left(n-1\right)+t^{2}\right]}\right) + \\ \frac{\mu_{\delta} t}{\sqrt{\frac{1}{2}\left(\frac{1}{n}+g\right)\left[\left(1+ng\right)\left(n-1\right)+t^{2}\right]}}\Gamma\left(\frac{n+1}{2}\right)\Bigg] \times \left[\frac{\frac{r}{\sqrt{2}}}{\Gamma(\frac{1}{2})}g^{-\frac{3}{2}}exp\left(-\frac{r^{2}}{2g}\right)\right]dg \end{aligned}}{\Gamma\left(\frac{n}{2}\right)\left(1+\frac{t^{2}}{n-1}\right)^{-\frac{n}{2}}}}   $$

where *n* is the sample size, *μ*_*δ*_ and *g* are the mean and standard deviation of the original effect size prior distribution, *t* is the *t*-test statistic, *Γ* denotes the Gamma function, _1_*F*_1_ denotes the confluent hypergeometric function, and *r* denotes the scale parameter of the Cauchy distribution. This expression allows making modifications to the prior distribution, such as increasing (decreasing) the scale parameter *r* for fields in which high effect sizes are more (less) frequent and shifting the center of the prior distribution away from zero for the implementation of Bayes factors in non-inferiority designs.

## Results

In the next subsections, we discuss calculating Bayes factors specifically for superiority and equivalence designs (for which the procedure is essentially identical) and non-inferiority designs. We provide worked examples of reanalyses of real data from publications of clinical trials for each of these to highlight the calculation of these Bayes factors, as well as to provide insight into the merits of this approach over more conventional analyses. Annotated code for conducting these reanalyses is available at https://osf.io/8br5g/.

### Bayes factors for superiority designs

For superiority designs, the null hypothesis is defined as *δ*=0. In order to evaluate this null hypothesis, we can use the Cauchy prior distribution for effect size *δ*, centered on zero. Ample examples of this approach have been reported elsewhere [12, 38]. Here, we will illustrate this approach with a reanalysis of data reported in [39].

#### Superiority of racemic adrenaline and on-demand inhalation with acute bronchiolitis

In Skjerven et al. [39], the authors examine the comparative efficacy of adrenaline inhalation by means of bronchodilators versus control (saline inhalations). Specifically, they test for superiority of racemic adrenaline over inhaled saline. In a separate hypothesis, the authors examine whether administration on a fixed schedule is superior to administration on demand. In both cases, the primary outcome is the length of stay in the hospital in hours. The authors conclude that “In the treatment of acute bronchiolitis in infants, inhaled racemic adrenaline is not more effective than inhaled saline. However, the strategy of inhalation on demand appears to be superior to that of inhalation on a fixed schedule.” The authors support their first conclusion with a *p*-value of.42 and their second conclusion with a *p*-value of.01. Note that the *p*-values reported by the authors suggest the performed tests were two-sided, although the study goals are more consistent with a one-sided test. In what follows, we report both a one- and two-sided reanalysis.

The reanalysis for the superiority test of racemic adrenaline over inhaled saline proceeds as follows:

- Obtain the standard error, *SE*_*treat*_, from the 95% confidence interval reported in Table 2 of Skjerven et al. [39]: *SE*_*treat*_=11/1.966≈5.6
- Calculate the *t*-statistic for the null-hypothesis that the difference in estimated length of stay between patients that inhaled racemic adrenaline and patients that inhaled saline is zero: $t = \frac {63.6-68.1}{5.6} = -0.80$ (which yields a two-sided *p*-value of.42).
- We use Eq. 4 to calculate a one-sided Bayes factor quantifying the relative likelihood of the one-sided alternative of superiority, *d*<0, versus the null hypothesis of no effect, *d*=0, given the data (*BF*_−0_). This leads to *BF*_−0_=0.24 (or *BF*_0−_=4.23), indicating that the null-hypothesis is over 4 times more likely than the one-sided alternative, given the data. The corresponding Bayes factor for a two-sided test is *BF*_10_=0.15 (or *BF*_0−_=6.64), indicating that the null-hypothesis is over 6 times more likely than the two-sided alternative, given the data.

These and other superiority Bayes factors can be obtained by providing values for the confidence interval margin, sample size n, and group means to the script: *CI*_*mar*_=(15.5−(−6.5))/2,*n*_1_=203,*n*_2_=201,*M*_1_=63.6, and *M*_2_=68.1 (further details can be found in the annotated code). This reanalysis corroborates the finding of the original authors, who found no significant difference between racemic adrenaline and inhaled saline. The Bayes factor indicates that the null hypothesis is a little over four times more likely than the one-sided alternative of superiority, given the data.

A similar reanalysis for the superiority of fixed schedule inhalation over inhalation on demand yields a one-sided Bayes factor *BF*_0−_=31.48 indicating that the null hypothesis is over 31 times more likely than superiority of fixed schedule inhalation over inhalation on demand, given the data. In the sample data, the trend is actually in the direction indicating superiority of inhalation on demand over fixed schedule inhalation. Given that the one-sided test compares two inappropriate hypotheses, we consider the results of a two-sided test more appropriate here. The Bayes factor in favor of a two-sided alternative, *BF*_10_, equals 2.24 (recall that [30] classify Bayes factors lower than 3 as not worth more than a bare mention). This finding tempers the conclusion of the original authors: although the data is slightly more consistent with the two-sided alternative hypothesis than with the null hypothesis, the Bayes factor suggests that the evidence is ambiguous and that more study is needed.

In sum, we have seen that Bayes factors can augment interpretation of the statistical evidence for superiority designs in important ways: we can quantify the strength of evidence of one hypothesis relative to another one; and we can explicitly quantify evidence in favor of the null hypothesis. The latter is particularly important for the evaluation of equivalence designs, to which we now turn.

### Bayes factors for equivalence designs

The objective of equivalence designs is to show that “the new treatment is at least as good as (no worse than) the existing treatment” [1]. Under a classical NHST approach, it is not possible to test for equivalence directly (the null hypothesis cannot be confirmed). As a result, equivalence needs to be tested by proxy by constructing a band around *δ*=0 of 2*c* and evaluating two null hypotheses: *δ*=−*c* and *δ*=*c*.

From a Bayesian perspective, the procedure is similar to that of the procedure for superiority designs. Instead of examining the Bayes factor’s strength of evidence in favor of *H*_1_, we now examine the strength of evidence in favor of *H*_0_. This removes the ambiguity associated with the traditional approach to equivalence testing. Examine for instance the example where equivalence was demonstrated in Fig. 1 (the fifth row). Equivalence was established, because both *δ*=−*c* and *δ*=*c* are rejected (i.e., the confidence interval lies fully between these two boundaries). However, the confidence interval does not overlap with *δ*=0, suggesting that the effect size is not zero, which is a counter-intuitive conclusion to draw simultaneously with the conclusion of equivalence.

Note that it is possible to calculate a Bayes factor for the same band around *δ*=0 of 2*c*, but there is no need as the evidence in favor of *δ*=0 can be quantified directly. Because of this, the Bayes factor approach simplifies testing for equivalence, such that no arbitrary band needs to be established. Furthermore, one is allowed to make claims about the absence of an effect, something that is not possible with the conventional NHST approach. We will illustrate this approach with a reanalysis of data reported in [40].

#### Equivalence between short- and long-term storage of red-cells on the Multiple Organ Dysfunction Score

In Steiner et al. [40], the authors examine the properties of the duration of storage for red-cells intended for transfusion. The authors assert that there is considerable uncertainty about potentially deleterious effects of long-term storage of red-cells before transfusion. In this study, the authors examine whether there are differences on the Multiple Organ Dysfunction Score (MODS) between patients that receive red-cells for transfusion that have been stored a short time (10 days or less) versus a long time (21 days or more). Although the authors do not explicitly conduct an equivalence design, the implicit goal seems to be to test whether or not longer storage of red cells is harmful. The authors conclude that “duration of red-cell storage was not associated with significant differences in the change in MODS”. The authors support this claim with a *p*-value of 0.44.

The application of the conventional NHST does not allow us to make any definite claims about the absence of a difference. In this demonstration, we reanalyze these data and calculate a Bayes factor to quantify the strength of evidence for equivalence provided by the data. For the analyses, we make use of the data presented in Table 2 of Steiner et al. [40]. In this table, the means are rounded to one decimal. To approximate the original analysis as accurately as possible, we work with means of 8.516 and 8.683 (reported means are 8.5 and 8.7, respectively) to approximate the reported *p*-value as closely as possible. For calculation of the Bayes factor, we assume a Cauchy prior centered on *δ*=0.

The reanalysis for the equivalence test of short- versus long-term storage of red-cells now proceeds as follows:

- Calculate the *t*-statistic for the null-hypothesis that the difference in MODS scores between patients that were administered red-cells that were stored short- versus long is zero: $t = \frac {8.516 - 8.683}{3.6\sqrt {1/538+1/560}} = -0.77$.
- We use Eq. 4 to calculate a two-sided Bayes factor quantifying the relative likelihood of the hypotheses *d*=0 versus *d*≠0 given the data (*BF*_01_). This leads to *BF*_01_=11.04, indicating that the null-hypothesis is over 11 times more likely than the two-sided alternative, given the data.

These and other equivalence Bayes factors can be obtained by providing values for the sample size n, group means, and group sds to the script: *n*_1_=538, *n*_2_=560, *M*_1_=8.516, and *M*_2_=8.683, *sd*_1_=3.6, and *sd*_2_=3.6 (further details can be found in the annotated code). This reanalysis corroborates the finding of the original authors, but allows us to go beyond the original claim by stating we have found evidence in favor of equivalence between short-term and long-term storage of red-cells as far as MODS scores are concerned. The Bayes factor lies between 3 and 20, which may be interpreted as positive evidence in favor of equivalence.

Steiner et al. [40] do not provide an equivalence margin, but it is important to stress that if they had, a Bayes factor for the relative likelihood of the population parameter being inside versus outside of this equivalence band can easily be calculated as well. Say, for instance, that *c*=0.05, then the two-sided Bayes factor quantifying the relative likelihood of the nul hypothesis −*c*<*d*<*c* versus the alternative hypothesis *d*<−*c* or *d*>*c* given the data is 19.09.

### Bayes factors for non-inferiority designs

**Fig. 2:**
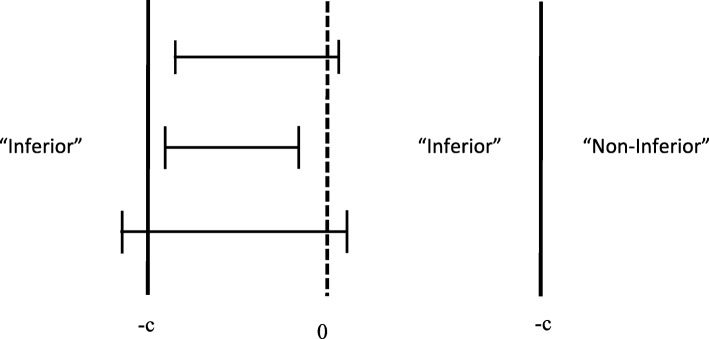
Non-inferiority design within an NHST framework (left) and a Bayesian framework (right). See text for details

In a traditional NHST approach, a non-inferiority design specifies a null-hypothesis of *δ*=−*c*, and requires a one-sided *z*- or *t*-test (or construction of a confidence interval). The crucial test is whether this test rejects or fails to reject inferiority (see Fig. 2, left panel).

The NHST approach for non-inferiority houses some unfortunate inconsistencies. Take for instance the top confidence interval in the left panel. This is an example of a situation where the null-hypothesis of inferiority gets rejected. From an NHST perspective, there is nothing wrong with this conclusion, as the confidence interval overlaps with zero, making the conclusion “non-inferior” warranted within that framework.

By contrast, examine the middle confidence interval in the left panel. Again, the null-hypothesis of inferiority gets rejected. This time, the implications are a bit less clear, because the confidence interval does not overlap with zero. From an NHST perspective, one would simultaneously reject the inferiority hypothesis and a classical one-sided null-hypothesis, reaching opposite conclusions. This makes it somewhat unclear if the conclusion “non-inferior” is really warranted here.

Finally, the bottom confidence interval in the left panel shows the scenario where the “inferior” null-hypothesis cannot be rejected. From an NHST perspective, we are unable to draw any further conclusions: is the drug/treatment inferior, or was the trial underpowered?

The Bayesian approach is not hampered by these pitfalls in interpretation. A Bayesian is concerned with the two hypotheses depicted in the right panel of Fig. 2. In [41] Bayesian approaches for non-inferiority trials are discussed (see also [42–44]), but discussion of the implementation of Bayes factors is limited to dichotomous data [45]. Here, we propose to calculate Bayes factors for continuous data, using the same principle as for superiority and equivalence designs illustrated above. The Bayes factor in this case quantifies the relative likelihood of the data having occurred given inferiority versus the likelihood of the data having occurred given non-inferiority.

Analogous to the Bayes factor for superiority/equivalence designs, we use the Cauchy prior distribution for effect size *δ*, centered on zero. The classical *z*- or *t*-statistics were evaluated against *δ*=−*c*. In order to maintain the theoretical property of the prior being centered on zero as specified in Jeffreys work, we shift the center of the Cauchy prior distribution to *c*. The easiest way to see why this is so is by imagining adding *c* to all data-points, all hypotheses, and all distributions, so that we evaluate the *t*-test for null hypothesis *δ*=0. The resulting test statistic will not change, as the data and the hypotheses have shifted by the same amount, but the prior distribution is now centered at *c*. Equation 4 allows for different specifications (for instance, a prior centered on the non-inferiority margin), but for these examples, we will keep the prior consistent across the design types. We will illustrate this approach with two examples. We first reanalyze dichotomous data published in [46], and then reanalyze continuous data published in [47].

#### Non-inferiority of beta-lactam

In [46], the authors examine antibiotic treatments for patients with clinically suspected community-acquired pneumonia (CAP). Specifically, guidelines recommend supplementing administration of beta-lactam with either macrolides or fluoroquinolones. The authors state that there is limited evidence that macrolides and/or fluoroquinolones have added benefits over the administration of just beta-lactam. In this study, the authors “tested the noninferiority of the beta-lactam strategy to the beta-lactam-macrolide and fluoroquinolone strategies with respect to 90-day mortality using a noninferiority margin of 3 percentage points and a two-sided 90% confidence interval.” The authors conclude that “the risk of death was higher by 1.9 percentage points (90% confidence interval [CI], -0.6 to 4.4) with the beta-lactam-macrolide strategy than with the beta-lactam strategy and lower by 0.6 percentage points (90% CI, -2.8 to 1.9) with the fluoroquinolone strategy than with the beta-lactam strategy. These results indicated noninferiority of the beta-lactam strategy.”

Sample sizes in the beta-lactam, beta-lactam-macrolide, and beta-lactam-fluoro-quinolone groups are 656, 739, and 888, respectively. The crude 90-day mortality was 9.0% (59 patients), 11.1% (82 patients), and 8.8% (78 patients), respectively, during these strategy periods. In this demonstration, we reanalyze these data and do two Bayesian tests for non-inferiority. For the analyses, we make the following assumptions:

- The critical non-inferiority tests compare two proportions. Like the original authors, we use the normal approximation for the sampling distribution of proportions. In all three groups, sample sizes are sufficiently large to make this a safe assumption.
- The Bayes factor approach requires specifying the non-inferiority margin in terms of effect size Cohen’s *d*. Cohen’s *h* for proportions has similar properties to Cohen’s *d* for continuous data. Converting the 3 percentage points yields a Cohen’s *h* of $2*\arcsin (\sqrt {\frac {59+82}{656+739}})-2*\arcsin (\sqrt {\frac {59+82}{656+739}-.03}) = 0.11$. Going forward, we will refer to this value as *h*.
- The equation we use to calculate the relevant Bayes factors, Equation 4, assumes a *t*-test statistic. For these sample sizes, the *t*-statistic is virtually indistinguishable from the *Z*-statistic provided by the normal approximation.

With these assumptions in place, the reanalysis for the beta-lactam versus beta-lactam-macrolide groups now proceeds as follows:

- Calculate the *Z*-statistic for the null-hypothesis that the difference in proportions of mortality in the beta-lactam group and the beta-lactam-macrolide group is.03: $Z = \frac {59/656-82/739-.03}{\sqrt {(59+82)/(656+739) \times (1-(59+82)/(656+739)) \times (1/656+1/739)}} = -3.16$.
- We use Eq. 4 to calculate a one-sided Bayes factor quantifying the relative likelihood of the hypotheses *h*<0.11 versus *h*=0.11 given the data (*BF*_−*h*_), and to calculate a one-sided Bayes factor quantifying the relative likelihood of the hypotheses *h*=0.11 versus *h*>0.11 given the data (*BF*_*h*+_).
- Finally, we use the principal of transitivity, *BF*_−+_=*BF*_−*h*_×*BF*_*h*+_. *BF*_−+_ quantifies the relative evidence for non-inferiority (difference in mortality rate is lower than 3 percentage points) versus inferiority (difference in mortality rate is higher than 3 percentage points), given the data. For these data, *BF*_−+_=1307.76, indicating that the non-inferiority hypothesis is over 1300 times more likely than the inferiority hypothesis, given the data.

These and other non-inferiority Bayes factors for proportions can be obtained by providing values for the sample size n, mortality count k, and the non-inferiority margin to the script: *n*_1_=656,*n*_2_=739,*k*_1_=59,*k*_2_=82, and *NI*_*mar*_=0.03 (further details can be found in the annotated code). A similar reanalysis for the beta-lactam versus beta-lactam-fluoroquinolone groups yields *BF*_−+_=39.07, indicating that the non-inferiority hypothesis is almost 40 times more likely than the inferiority hypothesis, given the data. Thus, our results corroborate those of the original authors, we find non-inferiority for beta-lactam versus beta-lactam-macrolide and beta-lactam-fluoroquinolone. The Bayes factors allow us to make claims about the strength of evidence, with support for non-inferiority of beta-lactam compared to beta-lactam-fluoroquinolone being strong, and support for non-inferiority of beta-lactam compared to beta-lactam-macrolide being overwhelming.

The above example demonstrates calculation of the Bayes factor for non-inferiority trials with dichotomous outcome measures. We now turn to a second example of our approach that showcases the application of our method for outcome data that is measured on a continuous scale.

#### Non-inferiority of internet-delivered cognitive behavior therapy

In [47], the authors examine the efficacy of internet-delivered cognitive behavior therapy (ICBT) in the treatment of mild to moderate depression symptoms, specifically by comparing its effectiveness to the ‘regular’ group-based cognitive behavior therapy (CBT). Depression symptoms are measured with the self-rated version of the Montgomery-Asberg Depression Rating Scale (MADRS). The authors define inferiority as a two-point difference on the MADRS between CBT and ICBT. The authors assess non-inferiority directly post-treatment and in a three-year follow-up and conclude that “Results on the self-rated version of the Montgomery-Asberg Depression Scale showed significant improvements in both groups across time indicating non-inferiority of guided ICBT.”

Sample sizes in the ICBT and CBT groups are 32 and 33 respectively post-treatment and 32 and 30 respectively in the three year follow-up. In this demonstration, we reanalyze these data and do two Bayesian tests for non-inferiority. For the analyses, we make use of the data presented in Table 2 of Andersson et al. [47].

The reanalysis for the ICBT versus CBT groups now proceeds as follows:

- The Bayes factor approach requires specifying the non-inferiority margin in terms of effect size Cohen’s *d*. Converting the 2 point difference yields a Cohen’s *d* of $d_{post} = 2/\sqrt {\frac {31*9.8^{2}+32*8^{2}}{63}} \approx 0.22$ for the post-treatment group and $d_{3} = 2/\sqrt {\frac {31*7.6^{2}+29*8.7^{2}}{60}}\approx 0.25$ for the three year follow-up group.
- Calculate the *t*-statistic for the null-hypothesis that the difference in MADRS scores in the ICBT group and the CBT groups is 2: $t_{post} = \frac {13.6 - 17.1 - 2}{\sqrt {\frac {31*9.8^{2}+32*8^{2}}{63}} \times \sqrt {1/32+1/33}} = -2.48$.
- We use Eq. 4 to calculate a one-sided Bayes factor quantifying the relative likelihood of the hypotheses *d*_*post*_<0.22 versus *d*_*post*_=0.22 given the data (*BF*_−*d*_), and to calculate a one-sided Bayes factor quantifying the relative likelihood of the hypotheses *d*_*post*_=0.22 versus *d*_*post*_>0.22 given the data (*BF*_*d*+_).
- Finally, we use the principal of transitivity, *BF*_−+_=*BF*_−*d*_×*BF*_*d*+_. *BF*_−+_ quantifies the relative evidence for non-inferiority (difference in depression scores is lower than 2 points) versus inferiority (difference in depression scores is higher than 2 points), given the data. For these data, *BF*_−+_=90.52, indicating that the non-inferiority hypothesis is over 90 times more likely than the inferiority hypothesis, given the data.

These and other non-inferiority Bayes factors for continuous data can be obtained by providing values for the sample size n, group means, group sds, and the non-inferiority margin to the script: *n*_1_=32,*n*_2_=33,*M*_1_=13.6, and *M*_2_=17.1,*sd*_1_=9.8,*sd*_2_=8, and *NI*_*mar*_=2 (further details can be found in the annotated code). A similar reanalysis for the three year follow-up non-inferiority test yields *BF*_−+_=353.61. Thus, our results corroborate those of the original authors, we find non-inferiority for ICBT versus CBT directly after treatment and in a three-year follow-up. Note that despite the relatively small sample size, the Bayes factors quantifying strength of evidence in favor of non-inferiority are substantial, highlighting one of the advantages of quantifying evidence with Bayes factors: a clear measure of the strength of evidence for one hypothesis relative to another that can be used to compare evidence across studies.

## Discussion

In this paper, we showed worked examples of the application of default Bayes factors to superiority, non-inferiority, and equivalence designs. In each of these cases, we believe that application of Bayes factors brings significant advantages. For superiority and equivalence designs alike, it is possible to explicitly quantify evidence in favor of the null hypothesis. For equivalence studies, specification of a potentially arbitrary band of equivalence is no longer necessary. For non-inferiority and equivalence designs alike, the interpretational hazard of simultaneously claiming non-inferiority/equivalence on one hand, but rejecting the null hypothesis of an effect size of zero on the other hand, disappears. The Bayes factor offers a way to quantify each of these types of evidence in a compelling and straightforward way.

**Fig. 3:**
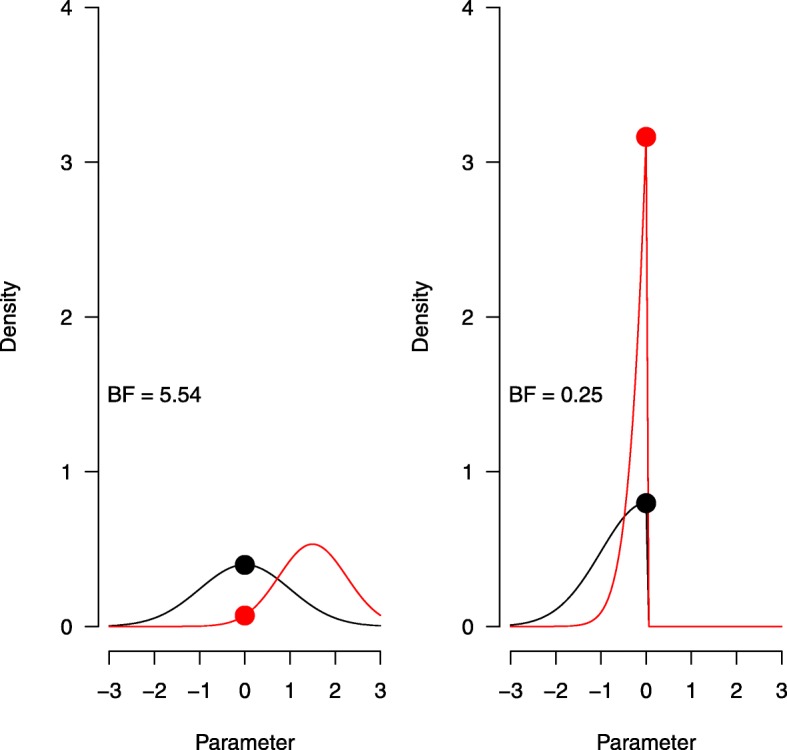
Hypothetical two-sided Bayes factor (left) and one-sided Bayes factor (right) for the same prior (black) and posterior (red) distribution on effect size

Some caveats to this kind of analysis should be considered. First of all, much like in NHST, one-tailed and two-tailed tests can give strikingly different results when the difference between groups is in the opposite direction of the one specified by the one-tailed test. We have seen such a scenario in our example for the superiority design, where we obtained *BF*_0−_=31.48 indicating that the null hypothesis is over 30 times more likely than superiority for our one-tailed test and *BF*_10_=2.24 indicating that the alternative hypothesis is slightly more likely than the null hypothesis for our two-tailed test. An illustration of how such discrepancies come about is given in Fig. 3.

The two panels demonstrate a two-sided Bayes factor (left) and a one-sided Bayes factor (right), calculated based on the same hypothetical prior (*N*(0,1)) and posterior (*N*(1,0.75)) distributions for effect size. In both cases, the Bayes factor is obtained by dividing the density of the prior, evaluated at zero, by the density of the posterior, evaluated at zero (i.e., the black dot divided by the red dot). For the one-sided Bayes factor, both distributions are truncated at zero. Because both distributions are normalized to have a density of 1, the effect of this truncation is especially strong for a distribution that falls almost entirely inside the truncated region, such as in the posterior distribution of our example data here and in the [39] superiority design data we reanalyzed. As a result, the Bayes factors in Fig. 3 lead to opposite conclusions, depending on whether the test was designed to be one-tailed or two-tailed. This example demonstrates that it is crucial to think about the hypotheses one wishes to test and the direction of testing before one obtains the data. Similar considerations apply when testing within the classical NHST framework.

Secondly, in NHST the status of *α*=0.05 is well established as a cut-off for significance (but see citeBenjaminEtAl2018). Bayesian inference does not have such universally agreed upon decision thresholds. Although different suggestions are offered in the literature [19, 30, 48], the authors caution against too rigid interpretation of these labels. We would argue that every cut-off value one chooses is to some extent arbitrary. With Bayes factors, one can at least choose a symmetrical cut-off score (for instance, we test until one hypothesis is 20 times more likely than the other given the data, so *BF*_10_=20 or *BF*_10_=1/20=0.05), whereas no such symmetry can be obtained with a *p*-value.

Thirdly, there are different ways to calculate Bayes factors [45]. Arguably the most important determinant for differences in Bayes factors stem from the choice of the underlying prior. Taking as an example the category of Bayes factors that assume a prior distribution on effect size, a prior that places a relatively high weight on an effect size of zero (i.e., is tightly peaked around zero), will lead to a relatively large Bayes factor in favor of the alternative hypothesis if the sample effect size is relatively different from zero. For reasonable priors, the effect of the choice of prior on the Bayes factor appears to be mostly quantitative and unlikely to alter the qualitative conclusions [31]. Nevertheless, in specific applications, these default prior analyses can be supplemented by substantive knowledge based on earlier experience. With a more informative prior distribution, the alternative hypothesis will make different predictions, and a comparison with the null hypothesis will therefore yield a different Bayes factor. The more informed the prior distribution, the more specific the model predictions, and the more risk the analyst is willing to take. Highly informed prior distributions need to be used with care, as they may exert a dominant effect on the posterior distribution, making it difficult to “recover” once the data suggest that the prior was ill-conceived. With informed prior distributions, it is wise to perform a robustness analysis to examine the extent to which different modeling choices lead to qualitatively different outcomes.

## Conclusions

Our paper offers an easy way of calculating Bayes factors for superiority, equivalence, and non-inferiority designs that is consistent across methods and scale of the outcome measure. With increasing accessibility of software aimed to conduct Bayesian inference [37], the absence of tools necessary to obtain Bayes factors is no longer a reason to refrain from using Bayesian analyses. We recommend standard consideration of Bayesian inference in clinical trials for obtaining strength of evidence that is consistent across studies.

## Abbreviations

BF: Bayes factor
CBT: Cognitive behavior therapy
CI: Confidence interval
ICBT: Internet-delivered cognitive behavior therapy
MADRS: Montgomery-Asberg depression rating scale
NHST: Null hypothesis significance test
